# Self-Reported Clinician Confidence in and Familiarity With Long COVID Care Following Continuing Medical Education Webinars: Unmatched Pre-Post Study

**DOI:** 10.2196/99790

**Published:** 2026-11-13

**Authors:** Vincent Zhong Xin, Samantha M R Kling, Sindhushree Hosakote, Ruth Adewuya, Cassandra Bragdon, Cati Brown-Johnson, Shelby Lazarow, Vivian Levy, Mitchell G Miglis, Leon S Moskatel, Amogha Paleru, Zara M Patel, Tom C Quach, Norah Simpson, Sarah Stranberg, Oliver R Sum-Ping, Phillip C Yang, Jonathan G Shaw, Hector Bonilla, Linda N Geng, Lauren Grossman

**Affiliations:** 1 Stanford Medicine, Stanford, CA, United States; 2 Evaluation Sciences Unit, Division of Primary Care and Population Health, Stanford Medicine, Stanford, CA, United States; 3 Division of Primary Care and Population Health, Stanford Medicine, Stanford, CA, United States; 4 Stanford Center for Continuing Medical Education, School of Medicine, Stanford Medicine, Stanford, CA, United States; 5 Division of General Psychiatry and Psychology, Stanford Health Care, Stanford, CA, United States; 6 San Mateo Medical Center, San Mateo County Health, Stanford Medicine, San Mateo, CA, United States; 7 Stanford Neuroscience Health Center, Faculty of Medicine, Stanford Medicine, Palo Alto, CA, United States; 8 Department of Neurology, Stanford Medicine, Stanford, CA, United States; 9 Department of Otolaryngology, Head and Neck Surgery, Stanford Medicine, Stanford, CA, United States; 10 Department of Psychiatry and Behavioral Sciences, Stanford Medicine, Stanford, CA, United States; 11 Outpatient Neurorehabilitation, Stanford Health Care, Stanford, CA, United States; 12 Stanford Center for Sleep Sciences and Medicine, Department of Psychiatry and Behavioral Sciences, Stanford Medicine, Stanford, CA, United States; 13 Department of Cardiovascular Medicine, Stanford Medicine, Stanford, CA, United States; 14 Department of Infectious Diseases, Faculty of Medicine, Stanford Medicine, Stanford, CA, United States; 15 Department of Primary Care and Population Health, Faculty of Medicine, Stanford Medicine, Stanford, CA, United States; 16 Department of Primary Care and Population Health, Faculty of Medicine, Stanford Medicine, 3351 El Camino Real, Ste 225, Atherton, CA, 94027, United States, 1 720-384-3454

**Keywords:** long COVID, postacute sequelae of SARS-CoV-2, continuing medical education, continuing professional development, virtual education, online learning, internet-based intervention, health personnel, physicians, program evaluation, health knowledge, self-efficacy

## Abstract

**Background:**

An estimated 18‐23 million Americans have had long COVID (LC), yet 54% and 72% of US physicians reported not being confident in diagnosing and treating LC, respectively. Continuing medical education (CME) is crucial to maintaining and improving clinical skills, and CME that incorporates need-based educational methods is associated with improved patient outcomes. Given limited clinician familiarity with LC, there is a need for an effective, widely accessible LC CME program.

**Objective:**

This study assessed the effectiveness of 2 Stanford University–organized LC CME webinars, which aimed to provide comprehensive, up-to-date evaluation and management strategies through multidisciplinary didactic presentations and question-and-answer sessions.

**Methods:**

Data from webinar attendees were collected through CME registration and prewebinar/postwebinar surveys. Registration surveys consisted of multiple-choice questions on profession, practice setting, experience treating vulnerable populations, and perception of LC. Prewebinar/postwebinar surveys consisted of multiple-choice questions on perception of LC, confidence in diagnosing LC, and familiarity with LC care. Prewebinar/postwebinar responses were compared using logistic regression, with Bonferroni adjustment to account for multiple testing. Statistical significance was defined as *P*<.05.

**Results:**

The August 2024 webinar had 449 registrants: 48.6% (n=218) were physicians, and 29.0% (n=130) reported an affiliation with an academic medical center. The attendee count was 248, with 116 and 90 attendees completing the prewebinar and postwebinar surveys, respectively. The proportion of respondents indicating that LC is a very or somewhat serious problem did not significantly differ between the pre-CME (93/115, 80.9%) and post-CME (75/88, 85.2%) surveys (adjusted *P*>.99). There were significant pre-to-post increases in the proportion of respondents indicating they were very confident in their LC diagnosis ability (11/102, 10.8% to 25/79, 31.6%; adjusted *P*=.004) and very familiar with LC care (10/91, 11.0% to 22/75, 29.3%; adjusted *P*=.02). The May 2025 webinar had 468 registrants: 52.6% (n=246) were physicians, and 24.8% (n=116) identified an academic medical center affiliation. The attendee count was 264, with 135 and 119 attendees completing the prewebinar and postwebinar surveys, respectively. The proportion of respondents indicating that LC is a very or somewhat serious problem did not significantly differ between the pre-CME (105/134, 78.4%) and post-CME (102/118, 86.4%) surveys (adjusted *P*=.49). There was a significant pre-to-post increase in the proportion of respondents indicating they were very familiar with LC care (12/116, 10.3% to 60/111, 54.1%; adjusted *P*<.001).

**Conclusions:**

CME participation was associated with significant increases in the proportions of respondents reporting confidence in diagnosing LC and familiarity with LC care, highlighting the potential of virtual CME programs to support clinician education.

## Introduction

Long COVID (LC), also known as postacute sequelae of SARS-CoV-2 (PASC), is a heterogeneous condition characterized by new or persistent symptoms that emerge or continue after acute SARS-CoV-2 infection and last at least 3 months [1,2]. LC can involve multiple organ systems and is associated with varying degrees of functional impairment and reduced quality of life [3].

It is estimated that between 18 and 23 million Americans have developed LC to date, with studies indicating that approximately 30% of individuals with acute COVID-19 infection report persistent symptoms months after the illness [4-8]. Despite this burden, in a 2022 poll of 806 US physicians, 54% reported not being confident in diagnosing LC, and 72% reported not being confident in treating it [9]. Clinician uncertainty may be compounded by the absence of a definitive diagnostic biomarker, heterogeneous symptoms that overlap with other chronic and postinfectious conditions, and the lack of a specific pharmacologic therapy [10].

The need for additional physician education on LC is further reflected in reports of underdiagnosis and unmet clinical needs from patients. In a study of 334 individuals with LC, 34% reported having their symptoms dismissed by medical professionals, 39% reported delays in accessing care, and 41% cited clinicians’ limited knowledge and inability to offer effective treatment [10,11]. In a separate survey study of 966 participants who self-identified as having LC, 95% reported experiencing stigma at least sometimes, and 76% reported experiencing stigma often or always [12].

Continuing medical education (CME) plays a crucial role in maintaining and improving the knowledge and skills of health care professionals [13-15]. In recent years, the adoption of online CME has accelerated due to the COVID-19 pandemic, with many in-person learning activities being converted to virtual formats [16]. Reported advantages of digitized CME include greater convenience, reduced travel and cost barriers, and the availability of recorded content for review [17]. Evaluations of online CME suggest that, when designed with sound educational principles, digital learning can achieve outcomes comparable to in-person instruction and support practice change [17-21].

Additionally, evidence from the CME literature suggests that multimodal and learner-engaged approaches are more likely to improve learning and translation into practice [14,15,22-26]. Technology-enabled CME can support these approaches by broadening reach and creating feasible, scalable opportunities for learner engagement, such as live question-and-answer (Q&A) sessions, virtual simulations, and chat-based discussions [16].

For LC, a condition that emerged in 2020 and for which clinical guidance continues to evolve, there is a need for practitioner-oriented CME that can efficiently reach clinicians across specialties and practice settings. Building on the existing literature, we developed 2 Zoom-based CME webinars on LC that aimed to address clinician knowledge gaps by providing up-to-date approaches to diagnosis and management through presentations by multidisciplinary faculty, with time allocated for participants’ questions and clarification.

## Methods

### Overview

We conducted a pre-post study of unmatched, deidentified attendee survey responses to evaluate changes in self-reported confidence and familiarity associated with 2 CME Zoom webinars on LC organized by the Stanford Center for Continuing Medical Education.

### Ethical Considerations

This study was approved by the Stanford University Institutional Review Board (IRB protocol #71861). Informed consent was not required because the research team received only deidentified secondary-use data.

### Participants

The webinars, titled *Unraveling Long COVID: Advances in Clinical Practice* and *Unraveling Long COVID: Clinical Advances & Future Directions*, were held on August 22, 2024, and May 29, 2025, respectively, from 9 AM to 1 PM Pacific Time, and were promoted via targeted email announcements, partner organization listservs, and professional networks. Health professionals were invited to register at no cost through the Stanford CME website. Participants were eligible to receive up to 3.25 and 3.00 credit hours, respectively, from the American Medical Association, American Nurses Credentialing Center, California Board of Registered Nursing, American Academy of Physician Associates, American Psychological Association, and American Board of Internal Medicine. In addition, participants in the May 2025 webinar were eligible to receive 3.00 continuing education units in physical therapy, occupational therapy, and speech-language pathology.

### Registration Data

At registration, all registrants were required to indicate their profession, report their ZIP code, and answer the following project-specific questions: (1) “What is your primary practice setting or affiliation?”; (2) “What estimated proportion of your clinical practice includes patients who are considered vulnerable or medically underserved populations?”; and (3) “How serious of a problem do you think Long COVID is in your patient population or practice setting?” Vulnerable or medically underserved populations were defined as “individuals who have poor access to health care and experience poor outcomes often as a result of factors such as poverty, race and ethnicity, language, health literacy, location, disabilities, gender, or sexual orientation” for the registrants. These questions were developed for this project by the clinical and evaluation team to understand attendees’ clinical setting and practice. Response data were collected through the Stanford CME Office registration platform. Descriptive statistics were applied to all responses to the registration form; no inclusion or exclusion criteria were applied. Registrant-entered ZIP codes were mapped using ArcGIS Online through the Stanford Geospatial Center, with invalid entries excluded. Descriptive statistics were used to describe the registrants; counts and percentages are reported. Analyses were conducted using SAS (version 9.4; SAS Institute Inc).

### Webinars

The educational objective of the webinars was to provide up-to-date strategies for diagnosing and managing LC via presentations from Stanford Health Care specialists in infectious diseases, cardiology, pulmonology, otolaryngology, neurology, gastroenterology, hematology, psychiatry, psychology, speech-language pathology, physical therapy, sleep medicine, and integrative medicine. Faculty were invited based on subject-matter expertise, clinical and research experience in LC, and ability to communicate with broad audiences of practicing clinicians. Presentations were 15 minutes each, and covered LC topics aligned with each speaker’s area of expertise, and were refined with input from the Stanford Center for CME to ensure balanced perspectives across disciplines and comprehensive coverage of priority themes. Each speaker’s content was designed to address current evidence and real-world challenges in LC care, focusing on practical clinical education by drawing on both prior research and frontline clinical experience. The breadth of topics covered reflects the complexity of LC and the multidisciplinary expertise required to address its diverse associated conditions. The differences in content between the 2 webinars enabled each program to be offered in a half-day format.

The August 2024 webinar also featured a performance by Joshua Roman, a cellist and LC patient advocate, who recounted his personal journey of healing and rediscovery in the face of LC, while the May 2025 webinar included a presentation on physical therapy presented by 2 clinicians from the University of North Carolina School of Medicine. Additionally, each webinar included 2 dedicated live Q&A sessions. The general structure and specific components of both webinars are detailed in Figure 1 and Table 1, respectively. The virtual setting and free registration were features designed to improve access to expert consultation and encourage attendance and participation by clinicians in the United States and abroad, including those from underserved areas.

**Figure 1.**
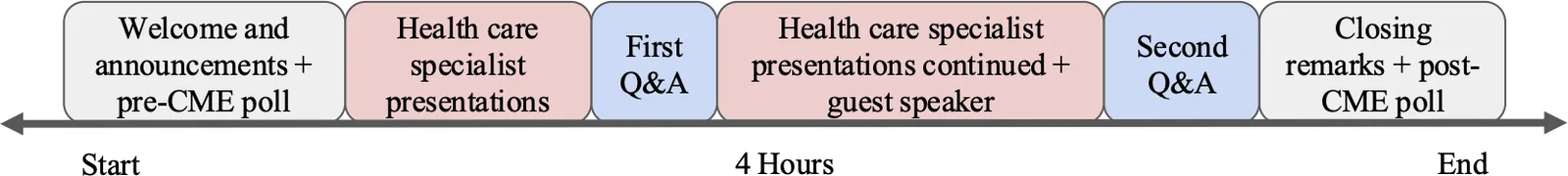
Stanford long COVID continuing medical education (CME) webinar structure. Q&A: questions and answers.

**Table 1.** Content differences between the August 2024 and May 2025 webinars.

CME^a^ component	August 2024	May 2025
Q&A^b^ Session	✓	✓
Long COVID patient performance and personal account	✓	
Didactic presentation: challenges of the diagnosis and equity of long COVID care	✓	
Didactic presentation: understanding myalgic encephalomyelitis and chronic fatigue syndrome overlap with long COVID	✓	
Didactic presentation: burden and impact of long COVID autonomic symptoms	✓	
Didactic presentation: post-COVID cardiac manifestations	✓	
Didactic presentation: impact of vaccines on long COVID symptoms	✓	
Didactic presentation: post-COVID pulmonary symptoms and management	✓	
Didactic presentation: sleep issues in long COVID	✓	
Didactic presentation: management of smell and taste loss post-COVID		✓
Didactic presentation: care for headache types in long COVID		✓
Didactic presentation: gastrointestinal issues post-COVID and their management		✓
Didactic presentation: coagulation and hematologic considerations in long COVID		✓
Didactic presentation: behavioral health management in long COVID		✓
Didactic presentation: coping with the psychological impact of long COVID		✓
Didactic presentation: neurocognitive rehabilitation and speech-language pathology for long COVID		✓
Didactic presentation: physical therapy and rehabilitation management in long COVID		✓
Didactic presentation: integrative and lifestyle medicine approaches for long COVID care		✓

a CME: continuing medical education.

b Q&A: questions and answers.

### Q&A Sessions

Each Q&A session was facilitated by a moderator who monitored the Zoom chat for participant questions, consolidated similar questions, and ensured that questions about the presentation topics were raised and addressed. Questions that could not be addressed during the live Q&A sessions were answered by faculty via the Zoom Chat so that no questions were missed.

### Presurvey and Postsurvey Questions

Survey questions were developed for this project by the clinical and evaluation team to assess the intended outcomes of the webinar: self-reported confidence in and familiarity with LC care. The surveys included the following questions: (1) “How serious of a problem do you think Long COVID is in your patient population or practice setting?” (response options: “Very serious problem,” “Somewhat serious problem,” “Not very serious problem,” “Not a problem,” and “Uncertain”); (2) “How confident do you feel in your ability to recognize and diagnose Long COVID?” (response options: “Very confident,” “Somewhat confident,” and “Not at all confident”); and (3) “How familiar are you with the current treatment and management options for Long COVID?” (response options: “Very familiar,” “Somewhat familiar,” and “Not at all familiar”). The August 2024 webinar Zoom polls included all 3 questions, but in the May 2025 webinar Zoom polls, the question “How confident do you feel in your ability to recognize and diagnose Long COVID?” was inadvertently omitted.

Zoom polls were sent to all live attendees at the beginning and end of each Zoom webinar. Participants were allotted approximately 2 minutes to complete each poll and were allowed to leave questions blank. Completion of the polls was not required to receive continuing education credit. Visual overviews of the number of registrants, attendees, poll respondents, and question respondents for each webinar are depicted in the *Results* section.

### Survey Data Collection

Survey responses were collected and deidentified by the Stanford Center for CME before analysis. The research team received only deidentified secondary-use data and had no access to identifiers or linking information. Consequently, for each webinar, poll data were aggregated without matching individual responses from before and after the webinars. All responses submitted by webinar attendees before the polls closed were included in the final dataset. No *a priori* sample size calculation was performed; the analytic sample included all available responses submitted before each poll closed.

### Survey Analysis

Responses to each Likert-scale question were dichotomized into two categories for analysis. For the question “How serious of a problem do you think Long COVID is in your patient population or practice setting?,” “How serious of a problem do you think Long COVID is in your patient population or practice setting?,” responses were grouped as “Very serious problem” and “Somewhat serious problem” vs “Not very serious problem,” “Not a problem,” and “Uncertain.” For the question “How confident do you feel in your ability to recognize and diagnose Long COVID?,” “How confident do you feel in your ability to recognize and diagnose Long COVID?,” responses were grouped as “Very Confident” vs “Somewhat Confident” and “Very Confident.” For the question “How familiar are you with the current treatment and management options for Long COVID?,” “How familiar are you with the current treatment and management options for Long COVID?,” responses were grouped as “Very familiar” vs “Somewhat familiar” and “Not at all familiar.”

Data from the August 2024 and May 2025 webinars were analyzed separately. For the August 2024 webinar, differences between the prewebinar and postwebinar responses were assessed using logistic regression with an LSMEANS statement and time (presurvey or postsurvey) as a fixed effect for each of the 3 questions (outcomes). Logistic regression was chosen because prewebinar and postwebinar responses were not linked at the participant level and were therefore analyzed as independent samples. For each question, all responses were included in the analyses.

For the May 2025 webinar, the same methodology was applied. Differences between the prewebinar and postwebinar responses were assessed using logistic regression with the LSMEANS statement, with time (presurvey or postsurvey) as a fixed effect for each of the 2 questions (outcomes). Prewebinar and postwebinar responses were not linked at the participant level and were therefore analyzed as independent samples. For each question, all responses to the question were included in the analyses.

Bonferroni adjustments were applied to account for multiple testing; both unadjusted and adjusted *P* values are reported. The Bonferroni family included 5 hypotheses testing whether the proportion of respondents selecting the desired response was higher in the postwebinar survey than in the prewebinar survey. The comparisons were: perceived seriousness, diagnostic confidence, and care familiarity for the August 2024 webinar; and perceived seriousness and care familiarity for the May 2025 webinar. Statistical significance was defined as a Bonferroni-adjusted *P*<.05. Estimated means and 95% CIs are reported. Analyses were conducted using SAS.

## Results

### August 2024 Webinar

A total of 449 people registered for the August 2024 webinar (Multimedia Appendix 1). Of these registrants, nearly half (n=218, 48.6%) were physicians and 29.0% (n=130) were affiliated with an academic medical center. Among the registrants, 68.4% (n=307) regarded LC as a very serious or somewhat serious problem in their practice setting. Table 2 presents a complete summary of responses to the August 2024 webinar registration questions.

**Table 2.** August 2024 webinar registrants’ profession, primary practice setting, and perceptions of long COVID as a problem (N=449).

Characteristics	August 2024 registrants, n (%)
Profession	
Physician	218 (48.6)
Nurse	65 (14.5)
Nonphysician	45 (10.0)
Student/trainee	43 (9.6)
Advance practice nurse	25 (5.6)
Behavioral/mental health	21 (4.7)
Allied health	15 (3.3)
Pharmacist	10 (2.2)
Industry/nonclinical	7 (1.6)
Primary practice setting	
Academic medical center	130 (29.0)
Private practice or medical group practice	87 (19.4)
Community or safety-net clinics and hospitals	81 (18.0)
Federal (Veterans Administration) or military health system	19 (4.2)
Other practice setting	61 (13.6)
Not applicable	71 (15.8)
Estimated percentage of practice with vulnerable populations	
0%‐25%	98 (21.8)
25%‐49%	79 (17.6)
50%‐75%	83 (18.5)
75%‐100%	83 (18.5)
Not applicable	106 (23.6)
Perceived seriousness of long COVID in practice setting	
Very serious problem	108 (24.1)
Somewhat serious problem	199 (44.3)
Uncertain	74 (16.5)
Not a very serious problem	55 (12.2)
Not a problem	13 (2.9)

In terms of geographic distribution, 269 out of the 449 (59.9%) registrants provided valid ZIP codes. As shown in Figure 2, these ZIP codes were distributed across the Americas, Europe, and Asia, with the highest concentrations observed in California and central Mexico. Smaller clusters were identified in France, Taiwan, and the northeastern United States.

**Figure 2.**
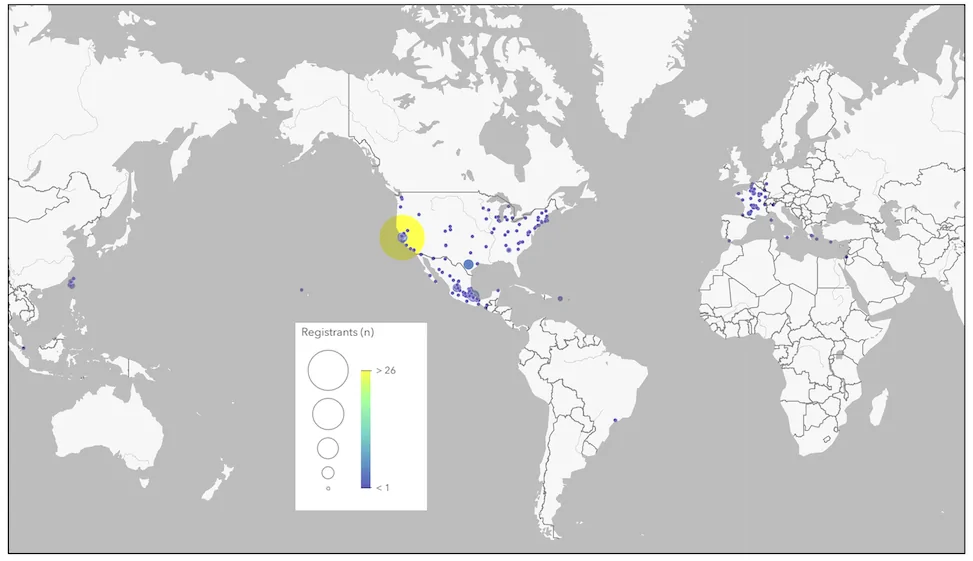
Geographic distribution of August 2024 webinar registrants by zip code (base map adapted from OpenStreetMap and its contributors; the underlying geographic data are licensed under the Open Data Commons Open Database License [ODbL] (CC BY-SA 2.0) by the OpenStreetMap Foundation [OSMF] [27]).

With respect to webinar participation, 248 out of the 449 (55.2%) registrants attended the final session. Among these attendees, 116 (46.8%) completed the prewebinar survey, and 90 (36.3%) completed the postwebinar survey (Figures 3 and 4). The number of responses varied between the prewebinar and postwebinar surveys and across questions, as participants were not required to answer all items. Item-level response rates varied by question. Among August prewebinar respondents (n=116), response rates were 99.1% (n=115) for seriousness, 87.9% (n=102) for confidence, and 78.4% (n=91) for familiarity (Table 3). Among August postwebinar respondents (n=90), response rates were 97.8% (n=88) for seriousness, 87.8% (n=79) for confidence, and 83.3% (n=75) for familiarity (Table 3). The distribution of responses for each question is shown in Figure 5 and Table 3.

**Figure 3.**
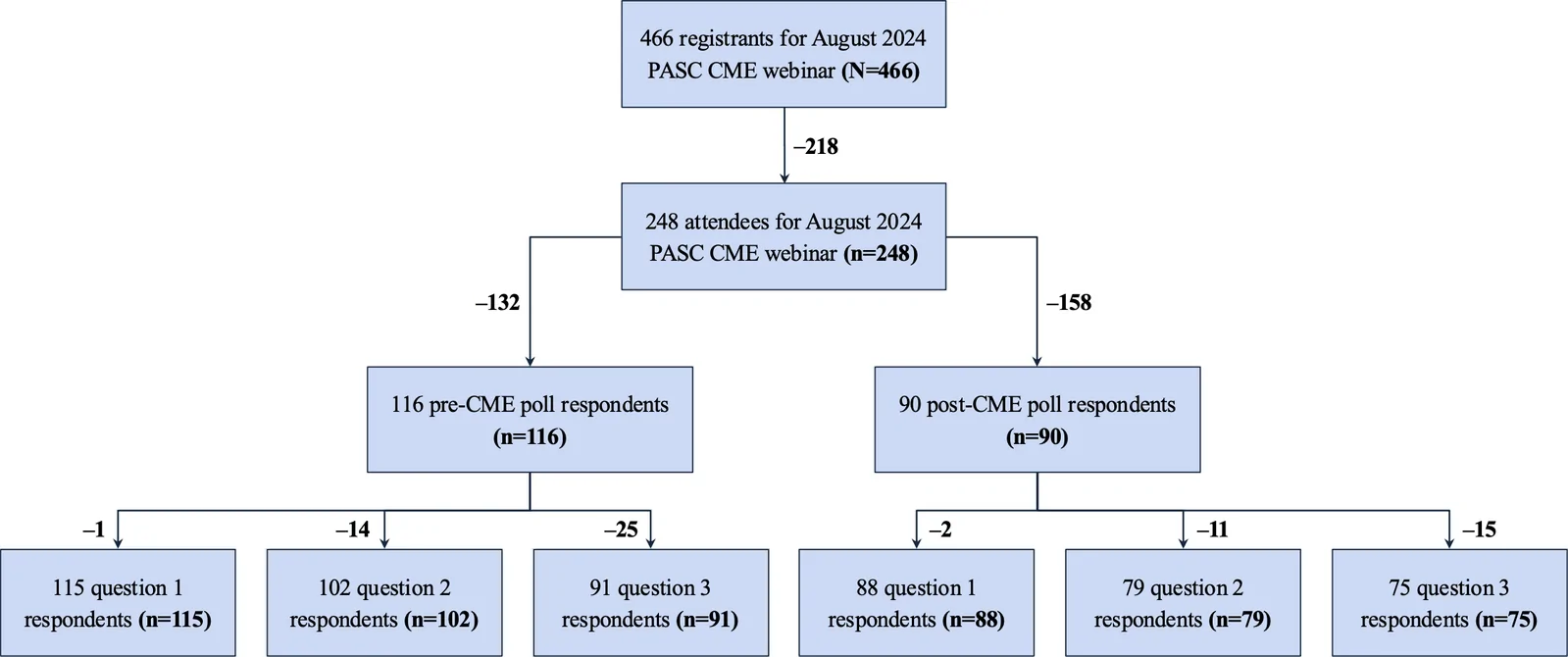
August 2024 webinar data collection flowchart. CME: continuing medical education; PASC: postacute sequelae of SARS-CoV-2.

**Figure 4.**
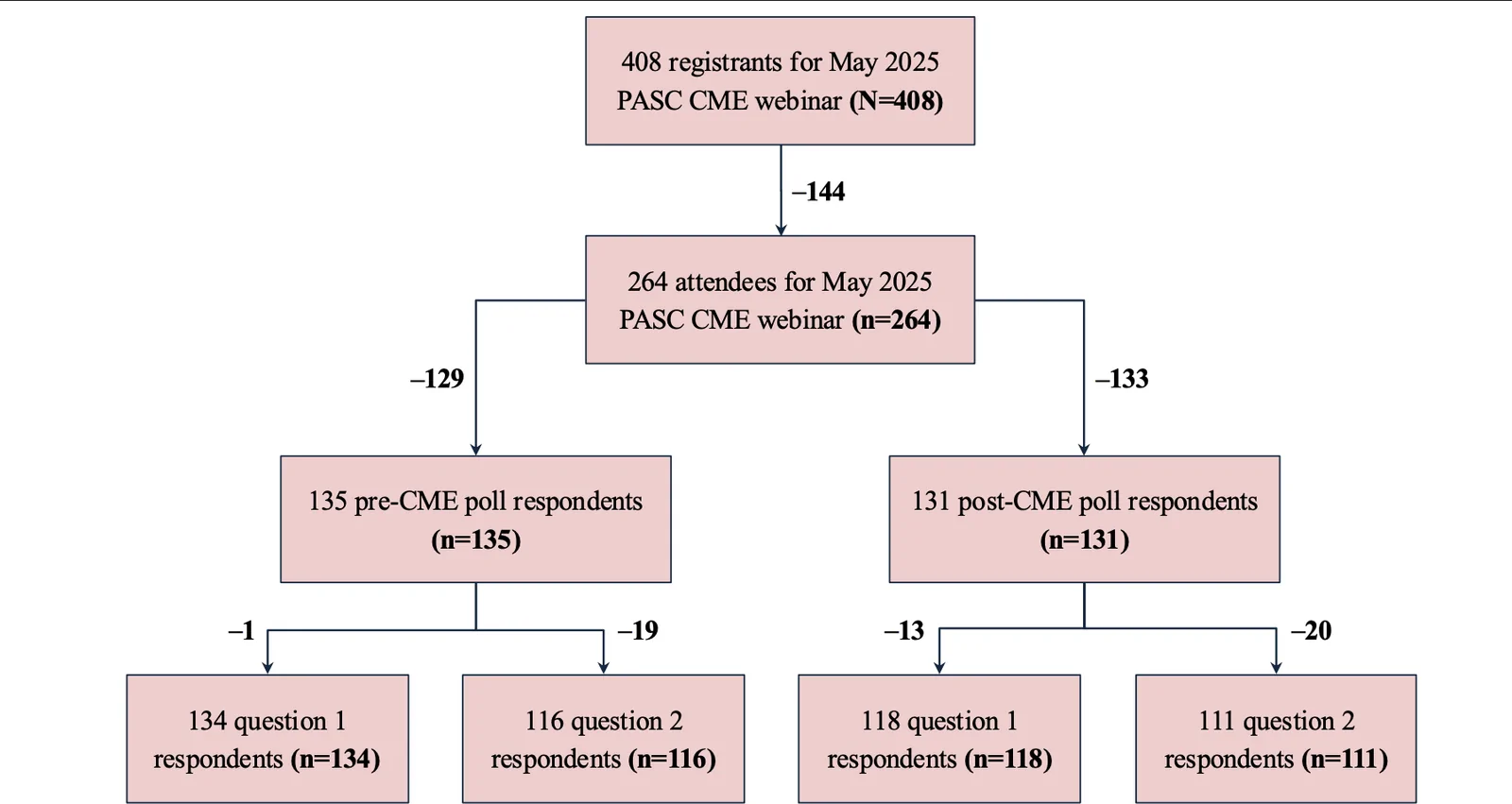
May 2025 webinar data collection flowchart. CME: continuing medical education; PASC: postacute sequelae of SARS-CoV-2.

**Table 3.** August 2024 continuing medical education (CME) webinar survey data and statistical analyses (total attendees: 248).

Questions and results	Pre-CME, n/N (%)	Post-CME, n/N (%)
How serious of a problem do you think Long COVID is in your patient population or practice setting?		
Very serious problem	42/115 (36.5)	38/88 (43.2)
Somewhat serious problem	51/115 (44.3)	37/88 (42.0)
Not very serious problem	10/115 (8.7)	6/88 (6.8)
Not a problem	2/115 (1.7)	3/88 (3.4)
Uncertain	10/115 (8.7)	4/88 (4.5)
How confident do you feel in your ability to recognize and diagnose Long COVID?		
Very confident	11/102 (10.8)	25/79 (31.6)
Somewhat confident	54/102 (52.9)	52/79 (65.8)
Not at all confident	37/102 (36.3)	2/79 (2.5)
How familiar are you with the current treatment and management options for Long COVID?		
Very familiar	10/91 (11.0)	22/75 (29.3)
Somewhat familiar	41/91 (45.1)	52/75 (69.3)
Not at all familiar	40/91 (44.0)	1/75 (1.3)

**Figure 5.**
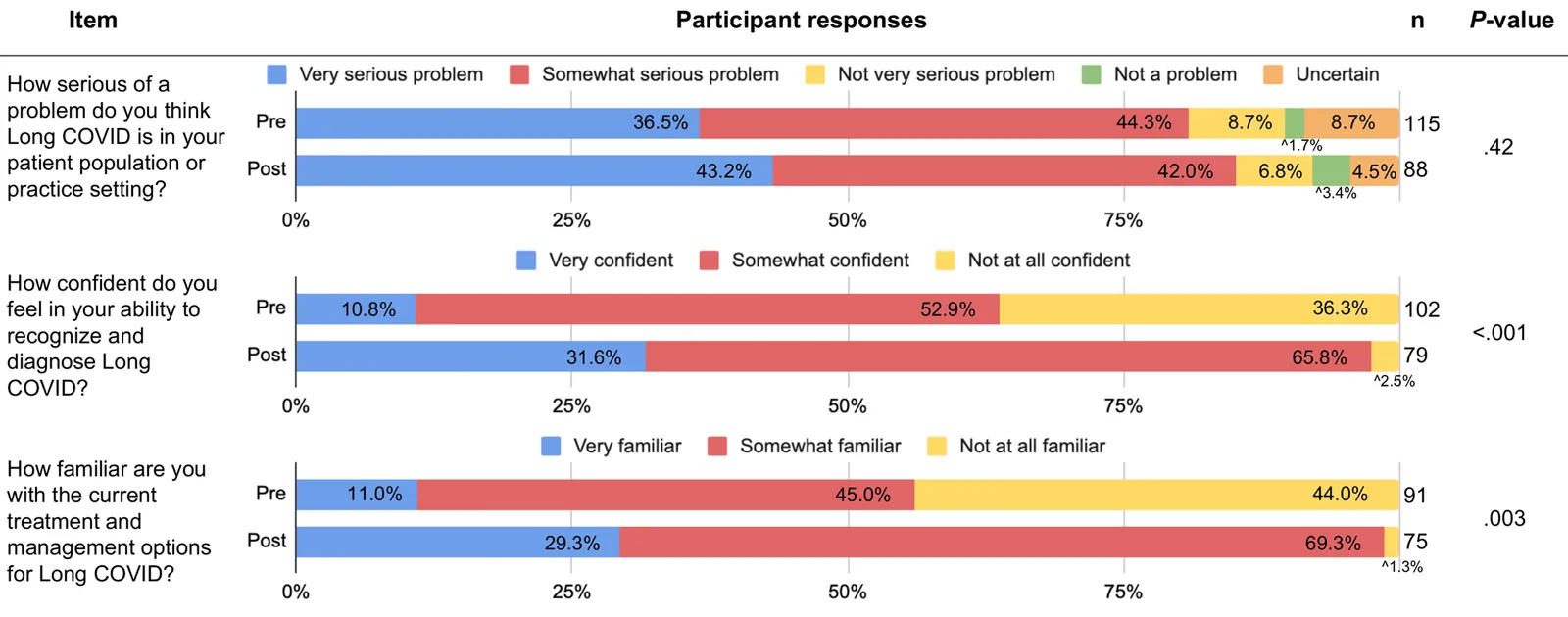
Participant survey response distributions at the beginning and end of the August 2024 CME webinar. CME: continuing medical education.

In response to the question, “How serious of a problem do you think Long COVID is in your patient population or practice setting?,” “How serious of a problem do you think Long COVID is in your patient population or practice setting?,” 80.9% (93/115) of prewebinar respondents and 85.2% (75/88) of postwebinar respondents reported that Long COVID was either a “very serious” or “somewhat serious” problem. The logistic regression indicated no significant difference between the predicted means for the prewebinar (80.9%, 95% CI 72.7%‐87.1%) and postwebinar (85.2%, 95% CI 76.2%‐91.2%) respondent groups (unadjusted *P*=.42; adjusted *P*>.99).

For the question, “How confident do you feel in your ability to recognize and diagnose Long COVID?,”“How confident do you feel in your ability to recognize and diagnose Long COVID?,” 10.8% (11/102) of prewebinar respondents reported feeling “very confident,” compared with 31.6% (25/79) of postwebinar respondents. Logistic regression analysis indicated a significant difference between the predicted means for the prewebinar (10.8%, 95% CI 6.1%‐18.4%) and postwebinar (31.6%, 95% CI 22.4%‐42.7%) respondent groups (unadjusted *P*=.001; adjusted *P*=.004).

Similarly, for the question, “How familiar are you with the current treatment and management options for Long COVID?,” “How familiar are you with the current treatment and management options for Long COVID?,” 11.0% (10/91) of prewebinar respondents reported being “very familiar,” compared to 29.3% (22/75) of postwebinar respondents. Logistic regression indicated a significant difference between the predicted means for the prewebinar (11.0%, 95% CI 6.0%‐19.2%) and postwebinar (29.3%, 95% CI 20.1%‐40.6%) respondent groups (unadjusted *P*=.004; adjusted *P*=.02)

### May 2025 Webinar

A total of 468 people registered for the May 2025 webinar (Multimedia Appendix 1). Of the registrants, over half (n=246, 52.5%) were physicians; 24.8% (n=116) were affiliated with an academic medical center; and 71.4% (n=334) regarded LC as a very serious or somewhat serious problem in their practice setting. Table 4 presents a complete summary of responses to the May 2025 webinar registration questions.

**Table 4.** May 2025 webinar registrant profession, primary practice setting, and perceptions of long COVID as a problem (total registrants: 468).

Characteristics	May 2025 registrants, n (%)
Profession	
Physician	246 (52.6)
Nurse	76 (16.2)
Nonphysician	32 (6.8)
Student or trainee	37 (7.9)
Advance practice nurse	31 (6.6)
Behavioral or mental health	18 (3.8)
Allied health	13 (2.8)
Pharmacist	3 (0.6)
Industry or nonclinical	4 (0.9)
Primary practice setting	
Academic medical center	116 (24.8)
Private practice or medical group practice	103 (22.0)
Community or safety-net clinics and hospitals	101 (21.6)
Federal (Veterans Administration) or military health system	9 (1.9)
Other practice setting	64 (13.7)
Not applicable	75 (16.0)
Estimated percentage of practice with vulnerable populations	
0%‐25%	113 (24.1)
25%‐49%	84 (17.9)
50%‐75%	95 (20.3)
75%‐100%	85 (18.2)
Not applicable	91 (19.4)
Perceived seriousness of long COVID in practice setting	
Very serious problem	111 (23.7)
Somewhat serious problem	223 (47.6)
Uncertain	68 (14.5)
Not a very serious problem	58 (12.4)
Not a problem	8 (1.7)

In terms of geographic distribution, 321 out of the 468 (68.6%) registrants provided valid ZIP codes, which are mapped in Figure 6. Consistent with the August 2024 webinar, registrant ZIP codes were distributed across the Americas, Europe, and Asia, with the highest concentrations observed in California and central Mexico. Smaller clusters were again identified in France, Taiwan, and the northeastern United States.

**Figure 6.**
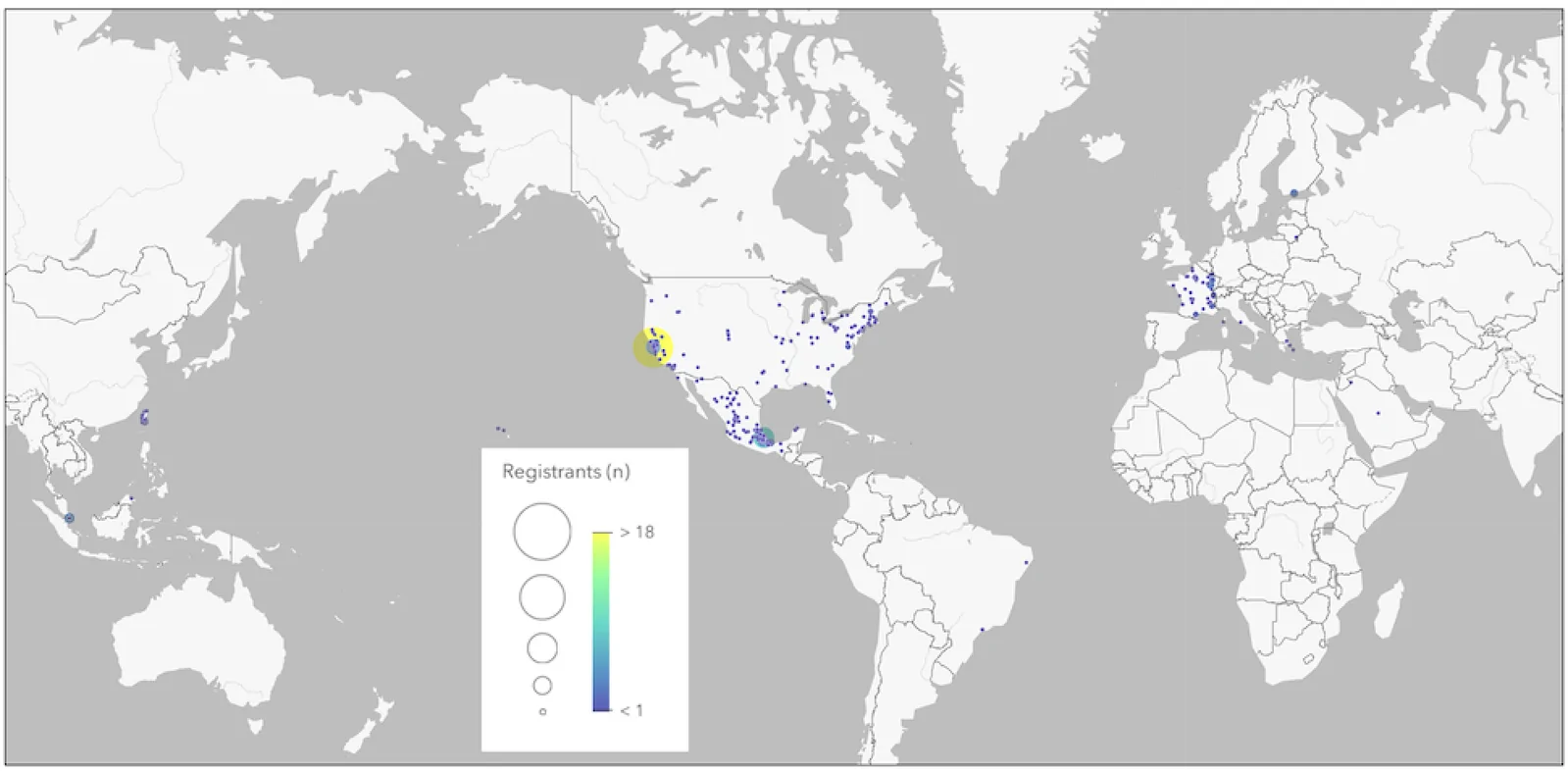
Geographic distribution of May 2025 webinar registrants by zip code (base map adapted from OpenStreetMap and its contributors; the underlying geographic data are licensed under the Open Data Commons Open Database License [ODbL] (CC BY-SA 2.0) by the OpenStreetMap Foundation [OSMF] [27]).

With respect to webinar participation, 264 out of the 468 (56.4%) registrants attended the final session. Among these attendees, 135 (51.1%) completed the prewebinar survey, and 119 (45.1%) completed the postwebinar survey. As with the August 2024 webinar, the number of responses varied by survey and by question due to optional participation. Item-level response rates varied by question. Among May 2025 prewebinar respondents (N=135), response rates were 99.3% (n=134) for seriousness and 85.9% (n=116) for familiarity (Table 5). Among May 2025 postwebinar respondents (N=119), response rates were 99.2% (n=118) for seriousness and 93.3% (n=111) for familiarity (Table 5). The distribution of responses for each question is shown in Figure 7 and Table 5.

**Table 5.** May 2025 continuing medical education (CME) webinar survey data and statistical analyses (total attendees: 264).

Questions and results	Pre-CME, n/N (%)	Post-CME, n/N (%)
How serious of a problem do you think Long COVID is in your patient population or practice setting?		
Very serious problem	42/134 (31.3)	46/118 (39.0)
Somewhat serious problem	63/134 (47.0)	56/118 (47.5)
Not very serious problem	17/134 (12.7)	7/118 (5.9)
Not a problem	1/134 (0.7)	4/118 (3.4)
Uncertain	11/134 (8.2)	5/118 (4.2)
How familiar are you with the current treatment and management options for Long COVID?		
Very familiar	12/116 (10.3)	60/111 (54.1)
Somewhat familiar	60/116 (51.7)	50/111 (45.0)
Not at all familiar	44/116 (37.9)	1/111 (0.9)

**Figure 7.**
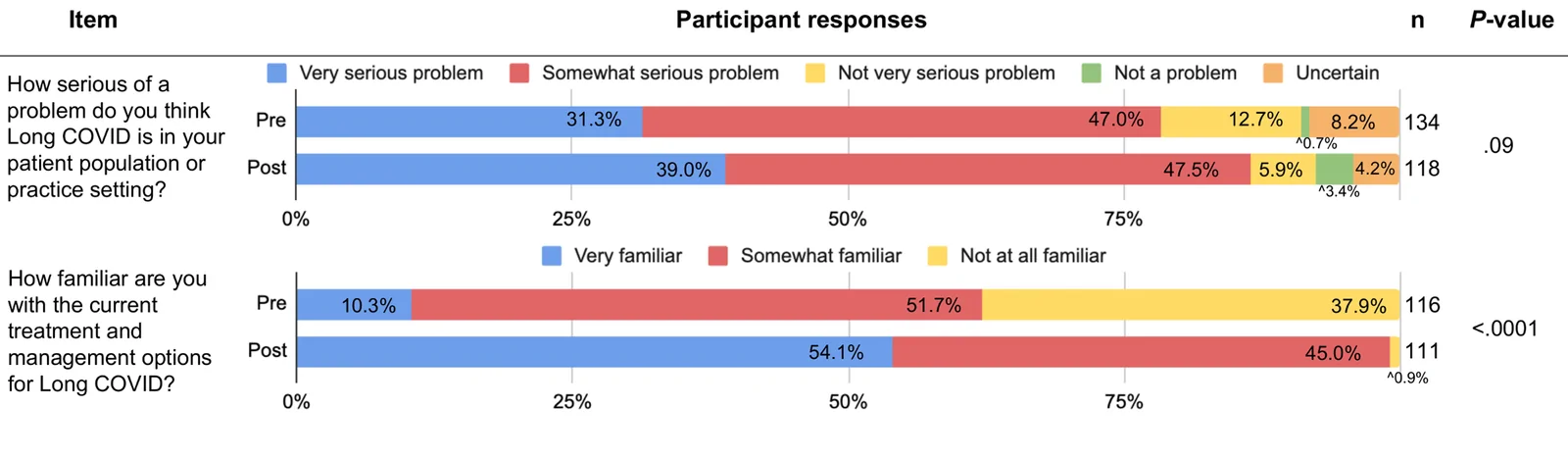
Participants’ survey response distributions at the beginning and end of the May 2025 CME webinar. CME: continuing medical education.

Regarding the perceived seriousness of LC, 78.4% (105/134) of prewebinar respondents and 86.4% (102/118) of postwebinar respondents reported that LC was a “very serious” or “somewhat serious” problem. The logistic regression indicated no significant difference between the predicted means of the prewebinar (78.4%, 95% CI 70.6%‐84.5%) and postwebinar (86.4%, 95% CI 79.0%‐91.5%) respondent groups (unadjusted *P*=.10; adjusted *P*=.49).

In terms of treatment familiarity, 10.3% (12/116) of prewebinar respondents indicated being “very familiar” with current treatment and management options, compared to 54.1% (60/111) of postwebinar respondents. The logistic regression indicated a significant difference between the predicted means for the prewebinar (10.3%, 95% CI 6.0%‐17.3%) and postwebinar (54.1%, 95% CI 44.8%‐63.1%) respondent groups (unadjusted *P*<.001; adjusted *P*<.001).

## Discussion

### Principal Results

This study found that participation in either of 2 live, multidisciplinary, 4-hour CME webinars on LC was associated with statistically significant increases in the proportion of clinicians who self-reported high diagnostic confidence and familiarity with treatment and management strategies. Prewebinar surveys indicated that relatively few participants felt “very confident” in diagnosing LC or “very familiar” with its management, mirroring prior national surveys documenting widespread uncertainty among US clinicians when faced with emerging syndromes such as LC [9].

Perceived seriousness of LC within participants’ practice settings was high across the registration, prewebinar, and postwebinar surveys, providing useful contextual information regarding clinicians’ perceptions of LC. Specifically, 68.4% (307/449) of August 2024 registrants and 71.4% (334/468) of May 2025 registrants rated LC as a “very” or “somewhat” serious problem at registration, compared with 80.9% (93/115) and 78.4% (105/134) in the prewebinar surveys and 85.2% (75/88) and 86.4% (102/118) in the postwebinar surveys, respectively.

Given that the August 2024 and May 2025 results are consistent with each other and with prior literature, these findings suggest that CME webinars may support clinician education on LC and increase self-reported confidence in diagnosis and familiarity with management among interested clinicians.

### Strengths and Limitations

Strengths of this study include the consistency of results from 2 nonoverlapping CME offerings. Additionally, the free, virtual format enabled participation from geographically and professionally diverse clinicians, increasing accessibility across the United States and internationally. Playlists containing recordings from both Zoom webinars were also uploaded to YouTube, with the August 2024 playlist receiving 9111 views and the May 2025 playlist receiving 5401 views. Furthermore, the multidisciplinary faculty presenters reflected the multisystem nature of LC, aligning educational content with the realities of clinical care.

This study had several limitations. First, as mentioned earlier, due to the voluntary nature of CME registration and survey completion, our sample is likely overrepresented by clinicians who were motivated to engage with the topic and perceive LC as a more serious problem than the average physician does. In addition, prewebinar and postwebinar responses were not linked at the participant level, and fewer respondents completed the postwebinar surveys. Participants could also leave individual survey items unanswered, resulting in item-level nonresponse and varying denominators across questions. Consequently, observed differences may partly reflect selective survey completion or differences in respondent composition rather than changes among individual participants. In particular, questions regarding diagnosis and treatment of LC may not have been applicable to attendees in nonclinical roles who do not diagnose patients or prescribe treatments, potentially contributing to item nonresponse. As such, our findings might not be fully generalizable to the broader health care professional population. Second, a large portion of our evaluation was based on self-reported data that did not extend past the end of either webinar, which introduces potential response bias and uncertainty about whether CME participation translates into sustained knowledge retention, behavior change, or improved patient outcomes. Third, as there was little overlap in didactic content between the 2 webinars and the survey items only assessed broad constructs of confidence and familiarity, we were unable to identify specific content or teaching methods that were the most impactful.

In addition, limitations inherent in the webinar medium may have affected both exposure and engagement: access depended on reliable internet, compatible devices, and user familiarity with the platform. The online format also afforded fewer opportunities for spontaneous discussion, small group case studies, and networking compared with in-person CME. Furthermore, the session was offered and captioned only in English, limiting accessibility and comprehension for clinicians with limited English proficiency.

### Implications and Future Directions

This work is particularly timely and important for advancing medical professionals’ understanding and management of LC, given that approximately 6% of US adults remain affected, of whom about one quarter report significant activity limitations [28]. More broadly, these results underscore the potential for well-designed, virtual CME to address urgent knowledge gaps in emerging disease areas.

Building on these findings, future CME efforts could implement longitudinal follow-up surveys to assess knowledge retention, collect objective measures of practice change, and compare outcomes between single and repeated exposures. Multilingual translations, captions, and adaptive learning platforms could also be incorporated to reach broader domestic and international audiences.

In a rapidly evolving clinical landscape, particularly for conditions like LC where the standard of care has yet to be established, CME should be nimble, evidence-based, and accessible. This study adds to the growing body of evidence supporting virtual, specialty-inclusive CME as a valuable tool for rapidly disseminating emerging clinical knowledge [16-21]. With thoughtful design and ongoing evaluation, such programs can help close the gap between evolving science and everyday patient care.

### Conclusion

Participation in 2 live, interdisciplinary CME webinars on LC was associated with significant increases in the proportions of respondents reporting high diagnostic confidence and familiarity with management strategies. These gains were consistent across both sessions despite differences in content and audience composition, underscoring the potential of well-designed virtual CME to address knowledge gaps in emerging conditions and the continued need for education on LC. LC was perceived as a serious clinical problem both before and after the CME sessions, likely reflecting high baseline awareness among participants. Overall, these findings support the use of accessible and specialty-inclusive CME as a strategy for rapidly disseminating evolving clinical knowledge and supporting clinician preparedness to care for patients with a complex, multisystem condition.

## Supplementary material

10.2196/99790 Multimedia Appendix 1 URL links to CME webinar YouTube playlists.

## Abbreviations

CME: continuing medical education
IRB: Institutional Review Board
LC: long COVID
PASC: postacute sequelae of SARS-CoV-2
Q&A: question-and-answer
