# Supplementary material for: Self-Reported Clinician Confidence in and Familiarity With Long COVID Care Following Continuing Medical Education Webinars: Unmatched Pre-Post Study
Source: JMIR Form Res. 2026 Nov 13;10:e99790. doi: 10.2196/99790 (PMC13766666; doi:10.2196/99790)
Supplement: Supplementary file 1 [file formative-v10-e99790-s001.docx]

Link to August 2024 CME Webinar YouTube Playlist: <https://youtube.com/playlist?list=PLuP5qOnCjJ-5oK3MNT8QGFNfTeVkm8-Nl&si=JFHdz-0LTgLwKO80>

Link to May 2025 CME Webinar YouTube Playlist: <https://youtube.com/playlist?list=PLuP5qOnCjJ-7o-RuABCmDLafA4ey06IS_&si=sq3vuO9dQkt15n5G>
